# Serum selenoprotein P, but not selenium, predicts future hyperglycemia in a general Japanese population

**DOI:** 10.1038/s41598-018-35067-2

**Published:** 2018-11-13

**Authors:** Swe Mar Oo, Hirofumi Misu, Yoshiro Saito, Mutsumi Tanaka, Seiji Kato, Yuki Kita, Hiroaki Takayama, Yumie Takeshita, Takehiro Kanamori, Toru Nagano, Masatoshi Nakagen, Takeshi Urabe, Naoto Matsuyama, Shuichi Kaneko, Toshinari Takamura

**Affiliations:** 1 Department of Endocrinology and Metabolism, Kanazawa University Graduate School of Medical Sciences, Kanazawa, Ishikawa Japan; 2 PRESTO, Japan Science and Technology Agency, Kawaguchi, Saitama Japan; 3 Department of Medical Life Systems, Faculty of Life and Medical Sciences, Doshisha University, Kyotanabe, Kyoto Japan; 4 Diagnostic R&D, R&D Headquarters, Alfresa Pharma Corporation, Ibaraki, Osaka Japan; 5 Department of Gastroenterology, Public Central Hospital of Matto Ishikawa, Matto, Ishikawa Japan; 6 Department of Gastroenterology, Kanazawa University Graduate School of Medical Sciences, Kanazawa, Ishikawa Japan

## Abstract

We aimed to test the hypothesis that selenoprotein P (SELENOP), a hepatokine involved in the development of both insulin resistance and impaired insulin production in mice, is related to future onset of hyperglycemia in humans. 76 healthy non-pregnant human subjects without diabetes underwent oral glucose tolerance test (OGTT) at baseline and 4-years follow-up. Nine subjects developed either impaired glucose tolerance or type 2 diabetes at follow-up. At baseline, SELENOP concentrations correlated negatively with insulinogenic index, but not with homeostasis model assessment-estimated insulin resistance (HOMA-IR). Multivariate analysis showed that baseline SELENOP predicted fasting plasma glucose at follow-up independently of the other parameters. The receiver operating characteristic (ROC) curve analysis showed that baseline concentrations of serum SELENOP, but not of selenium, were a reliable test to predict future onset of glucose intolerance. In conclusion, elevation of circulating SELENOP, but not of circulating selenium, was positively and independently associated with future onset of glucose intolerance in a general Japanese population.

## Introduction

We have rediscovered selenoprotein P (SELENOP) as a hepatokine that induces insulin resistance and hyperglycemia in mice^1^. SELENOP (encoded by the *SELENOP* gene in humans) is an abundant plasma protein primarily produced by the liver^2^. SELENOP, a secretory protein with 10 selenocysteine residues per polypeptide, is reported to function as a selenium transport protein^3,4^. The role for SELENOP in the regulation of glucose metabolism was formerly unknown, but SELENOP has thereafter emerged from human liver screening for secretory proteins whose hepatic gene expression levels are positively correlated with the severity of insulin resistance. Treatment with purified SELENOP impairs insulin signal transduction in both cultured cells and mice. These results raise the possibility that SELENOP functions as a hepatokine that leads to the onset of insulin resistance in mice under over-nutritional conditions^1^. More recently, we have shown that SELENOP impairs the health-promoting effects of exercise training by suppressing exercise-induced adaptations in the skeletal muscle via the receptor low-density lipoprotein receptor-related protein 1 (LRP1)^5^. These reports suggest that overproduction of SELENOP contributes to the development of various kinds of pathologies in life style-associated diseases such as type 2 diabetes^6,7^.

A number of previously-published studies have shown the inconsistent results regarding the relationship between circulating levels of SELENOP and metabolic disorders. First, SELENOP concentrations were reported to be elevated in people with type 2 diabetes, prediabetes, and non-alcoholic fatty liver disease^1,8,9^. However, pregnant women with gestational diabetes showed unchanged plasma levels of SELENOP compared with those without gestational diabetes^10^. In young children, increased SELENOP concentrations were reported to be negatively associated with certain components of metabolic syndrome, such as waist circumference and blood pressure^11^. A clinical study using magnetic resonance imaging reported negative correlations between serum SELENOP and visceral and subcutaneous fat volumes^12^. In contrast, another study found the opposite pattern, where plasma SELENOP was increased in people with obesity, but was not independently associated with the severity of insulin resistance^13^. In these papers described above, different commercially available enzyme-linked immunosorbent assay (ELISA) kits were used to measure blood concentrations of SELENOP. However, the recent report has shown that SELENOP values measured by some commercial ELISA kits did not correlate at all with those measured by our sol particle homogeneous immunoassay methods that are selective for measurement of full length form of SELENOP^14^. This paper suggests that the choice of ELISA kits is critical for discussing circulating levels of human SELENOP including the reproducibility of our previous results.

To date, there were no prospective studies that determine whether elevation of blood concentrations of SELENOP is linked to the future onset of hyperglycemia in humans. Based on our previous reports described above, we hypothesized that elevation of circulating SELENOP is connected to the future onset of hyperglycemia in humans. To test this hypothesis, we performed a prospective study that examines blood concentrations of SELENOP and glucose tolerance in Japanese non-pregnant people without diabetes who underwent a complete physical examination.

## Results

### Baseline and follow-up characteristics of glucose metabolism in human subjects

The baseline clinical and laboratory variables of 76 healthy human subjects are shown in Table 1. We analyzed parameters involved in glucose and selenium metabolism at two time points, baseline and 4-years follow-up (Table 2). All the subjects showed normal glucose tolerance at baseline, but 9 subjects developed either impaired glucose tolerance or type 2 diabetes at follow-up. Concentrations of fasting plasma glucose, 60 minutes plasma glucose, and 120 minutes plasma glucose increased significantly at 4-years follow-up. Insulinogenic index, a marker of early insulin secretion from pancreatic β-cells^15^, decreased significantly, whereas HbA1c and homeostasis model assessment-estimated insulin resistance (HOMA-IR)^16^ were unchanged before and after the follow up period. Among the parameters involved in selenium metabolism, concentrations of SLENOP and activity of glutathione peroxidase 3 (GPX3), another extracellular selenoprotein with anti-oxidative capacity^17^, increased before and after the follow-up period.

**Table 1:** Baseline clinical and laboratory variables.

Characteristics	Baseline
*n*	76
Age (years)	51.9 ± 10.5
Gender (M/F)	42/34
Body weight (kg)	60.1 ± 9.7
BMI (kg/m^2^)	22.8 ± 3.0
Waist circumference (cm)	80.8 ± 8.6
Systolic blood pressure (mmHg)	123.0 ± 17.4
Diastolic blood pressure (mmHg)	77.8 ± 10.6
AST (IU/L)	22.0 ± 6.1
ALT (IU/L)	22.1 ± 11.2
Triglyceride (mg/dL)	113.6 ± 72.5

Data are means ± SD. BMI, body mass index; HbA1c, glycosylated hemoglobin; HOMA-IR, homeostasis model assessment of insulin resistance; AST, aspartate aminotransferase; ALT, alanine aminotransferease.

**Table 2:** Clinical parameters involved in glucose and selenium metabolism at baseline and 4-year follow-up.

Characteristics	Baseline	4-year follow-up	*P*
*n*	76	76	
BMI (kg/m^2^)	22.8 ± 3.0	22.8 ± 2.9	0.945
Fasting plasma glucose (mg/dl)	92.5 ± 8.4	97.1 ± 9.6	<0.001**
Plasma glucose 30 min (mg/dl)	144.2 ± 25.2	149.3 ± 31.1	0.061
Plasma glucose 60 min (mg/dl)	131.3 ± 40.5	144.3 ± 45.2	0.004**
Plasma glucose 120 min (mg/dl)	109.0 ± 24.7	116.0 ± 26.0	0.022*
Fasting IRI (µU/ml)	5.7 ± 2.9	4.9 ± 2.6	0.025*
IRI 30 min (µU/ml)	47.9 ± 27.4	40.7 ± 33.5	0.141
IRI 60 min (µU/ml)	47.1 ± 24.8	46.6 ± 31.6	0.884
IRI120 min (µU/ml)	34.3 ± 20.4	33.0 ± 20.7	0.652
HbA1c (%)	5.2 ± 0.3	5.2 ± 0.3	0.763
HOMA-IR	1.31 ± 0.71	1.18 ± 0.67	0.152
Insulinogenic index	0.93 ± 0.69	0.69 ± 0.51	0.001**
SELENOP (μg/mL)	2.51 ± 0.52	3.81 ± 0.60	<0.001**
Selenium (μg/L) (*n* = 44)	157.9 ± 21.7	205.0 ± 22.1	0.075
GPX3 (U/L) (*n* = 44)	190.4 ± 68.5	238.3 ± 55.2	<0.001**

Data are means ± SD.

*p < 0.05, **p < 0.01.

BMI, body mass index; IRI, immunoreactive insulin; HbA1c, glycosylated hemoglobin; HOMA-IR, homeostasis model assessment of insulin resistance; SELENOP, selenoprotein P; GPX3, glutathione peroxidase 3.

### Circulating SELENOP, but not selenium, at baseline is positively associated with future hyperglycemia

**Figure 1:**
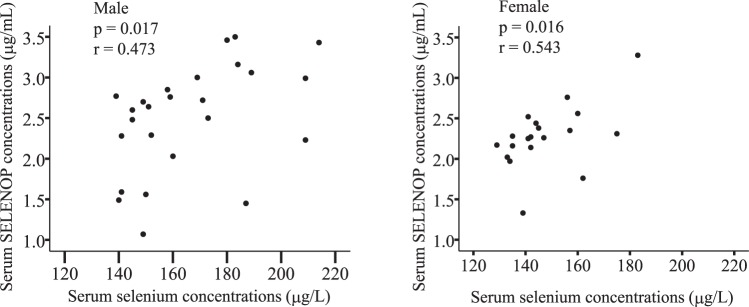
Correlation between serum concentrations of selenium and selenoprotein P at baseline in healthy people. Graphs show individual correlations between serum concentrations of selenium and selenoprotein P (SELENOP) in Japanese general population (*n* = 25 for males; *n* = 19 for females).

Linear regression analysis of baseline concentrations of SELENOP and selenium with different baseline clinical parameters is shown in Table 3. SELENOP concentrations at baseline positively correlated with age (*r* = 0.252, *P* = 0.028), but not with body weight, body mass index (BMI), waist circumference, liver functions and serum triglyceride concentrations. Among the parameters involved in glucose metabolism, no significant correlations were found between baseline serum SELENOP concentrations and fasting plasma glucose, fasting immunoreactive insulin, HOMA-IR or HbA1c. However, SELENOP concentrations were significantly associated with 30 minutes and 60 minutes plasma glucose concentrations in OGTT test (*r* = 0.241, *P* = 0.036, and *r* = 0.326, *P* = 0.004, respectively). Additionally, serum concentrations of SELENOP at baseline were negatively correlated with insulinogenic index, a marker of early insulin secretion from pancreatic β-cells^15^. In contrast, selenium concentrations showed no correlation with plasma glucose concentrations at baseline (Table 3), although serum concentrations of selenium positively correlated with those of SELENOP in both male and female participants (Fig. 1).

**Table 3:** Linear regression analysis of baseline selenoprotein P concentrations with clinical parameters at baseline.

Parameters at baseline	SELENOP concentration at baseline (*n* = 76)		Selenium concentration at baseline (*n* = 44)	
	*p* value	*r*	*p* value	*r*
Age	0.028*	0.252	0.001**	0.501
Body weight	0.177	−0.157	0.217	−0.190
BMI (kg/m^2^)	0.721	−0.042	0.225	0.187
Waist Circumference (cm)	0.844	−0.023	0.029*	0.329
Fasting plasma glucose (mg/dl)	0.092	0.194	0.102	0.250
Plasma glucose 30 min (mg/dl)	0.036*	0.241	0.716	0.056
Plasma glucose 60 min (mg/dl)	0.004**	0.326	0.373	0.138
Plasma glucose 120 min (mg/dl)	0.797	0.030	0.866	−0.026
Fasting IRI (µU/ml)	0.498	−0.079	0.694	0.061
IRI 30 min (µU/ml)	0.261	−0.130	0.046*	−0.302
IRI 60 min (µU/ml)	0.107	0.187	0.932	−0.013
IRI 120 min (µU/ml)	0.874	−0.018	0.216	−0.190
HbA1c (%)	0.331	0.113	0.571	0.088
HOMA-IR	0.723	−0.041	0.501	0.104
Insulinogenic index	0.010*	−0.293	0.070	−0.276
AST (IU/L)	0.737	−0.039	0.021	−0.347
ALT (IU/L)	0.472	−0.084	0.089	−0.259
Triglyceride (mg/dl)	0.970	0.004	0.426	−0.123

^*^p < 0.05, **p < 0.01.

BMI, body mass index; IRI, immunoreactive insulin; HbA1c, glycosylated hemoglobin; HOMA-IR, homeostasis model assessment of insulin resistance; AST, aspartate aminotransferase; ALT, alanine aminotransferease; SELENOP, selenoprotein P.

Then, we performed a linear regression analysis of baseline selenoprotein P concentrations with various clinical parameters at 4-years follow-up to examine whether circulating SELENOP is associated with future glucose homeostatic status in healthy subjects. As shown in Table 4, baseline SELENOP concentrations showed significant positive correlation with plasma glucose concentrations at all time points in OGTT at 4-years follow-up. Furthermore, a significant negative correlation was found between baseline SELENOP concentrations and insulinogenic index at 4-years follow-up (*r* = −0.296, *P* = 0.012). However, unlike SELENOP, selenium concentrations at baseline and the increment of selenium concentrations during the follow up period showed no correlation with plasma glucose concentrations at the 4-year follow-up (Table 4 and Supplementary Table 5). These data indicate strong association of baseline circulating SELENOP, but not of baseline selenium, with future glucose intolerance in this population.

**Table 4:** Linear regression analysis of baseline selenoprotein P concentrations with clinical parameters at the 4-year follow-up in all the participants.

Parameters at 4-year follow-up	SELENOP concentration at baseline (*n* = 76)		Selenium concentration at baseline (*n* = 44)	
	*p* value	*r*	*p* value	*r*
Body weight	0.401	0.098	0.040*	0.310
BMI (kg/m^2^)	0.855	−0.021	0.266	0.171
Fasting plasma glucose (mg/dl)	0.008**	0.303	0.223	0.187
Plasma glucose 30 min (mg/dl)	0.021*	0.264	0.484	0.108
Plasma glucose 60 min (mg/dl)	<0.001**	0.451	0.191	0.201
Plasma glucose 120 min (mg/dl)	0.045*	0.230	0.271	0.170
Fasting IRI (µU/ml)	0.075	−0.205	0.076	−0.270
IRI 30 min (µU/ml)	0.029*	−0.251	0.393	−0.132
IRI 60 min (µU/ml)	0.154	0.165	0.201	−0.196
IRI 120 min (µU/ml)	0.672	0.049	0.355	−0.143
HbA1c (%)	0.157	0.164	0.089	0.260
HOMA-IR	0.229	−0.140	0.138	−0.227
Insulinogenic index	0.012*	−0.296	0.090	−0.268

^*^p < 0.05, **p < 0.01.

BMI, body mass index; IRI, immunoreactive insulin; HbA1c, glycosylated hemoglobin; HOMA-IR, homeostasis model assessment of insulin resistance; SELENOP, selenoprotein P.

Next, we assessed the correlation between baseline SELENOP and plasma glucose at 4-years follow-up separately for male and female participants (Supplementary Tables 1, 2). In male participants, SELENOP concentrations at baseline correlated with the 60-minute plasma glucose concentration at the 4-year follow-up, whereas in female participants, SELENOP concentrations at baseline correlated with fasting plasma glucose at the 4-year follow-up.

### Baseline GPX3 is not positively associated with future hyperglycemia

We assessed the relationship between another extracellular selenoprotein GPX3 and future hyperglycemia. At baseline, GPX3 activity showed negative correlation with waist circumference and plasma glucose at 30 min, but no correlation with SELENOP no selenium concentrations (Supplementary Table 3). Unlike SELENOP, GPX3 activity at baseline did not show a correlation with plasma glucose concentrations at any time points over the 4-year follow-up (Supplementary Table 4).

### Baseline circulating SELENOP is an independent predictor of follow-up fasting plasma glucose concentrations in healthy people

To clarify the contribution of baseline serum SELENOP concentrations on the future risk of hyperglycemia, we generated multivariate linear regression models using plasma glucose concentration at 4-years follow up as a dependent variable. As shown in Table 5, baseline serum concentrations of SELENOP predicted fasting plasma glucose concentrations at 4-years follow-up independently of the other clinical parameters such as age, insulinogenic index, BMI and HbA1C.

**Table 5:** Multivariate regression analysis of fasting plasma glucose concentrations at 4-year follow-up as a dependent variable (*n* = 76).

Parameters at baseline	Fasting plasma glucose at 4-year follow-up		
	β	*p* value	Variance inflation factors
Age	0.027	0.816	1.215
Insulinogenic index	−0.164	0.141	1.108
SELENOP	0.237	0.033^*^	1.076
BMI	0.108	0.312	1.012
HbA1c	0.280	0.013^*^	1.091

^*^p < 0.05.

See Table 1 for abbreviations.

### Baseline SELENOP is the most reliable test to predict future glucose intolerance

**Figure 2:**
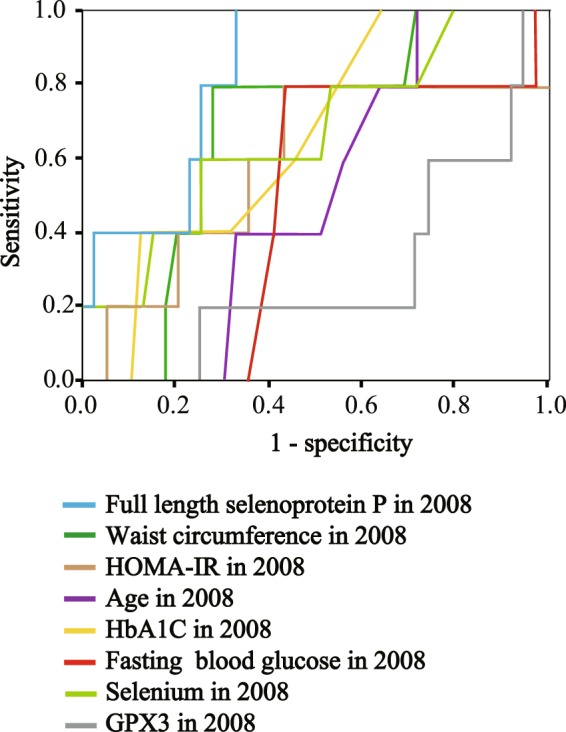
ROC analysis of the onset of glucose intolerance at 4-years follow-up in healthy people. Receiver operating characteristic (ROC) curve analysis of the sensitivity and specificity for the onset of glucose intolerance at 4-years follow-up by selenoprotein P (SELENOP) concentrations, waist circumference, HOMA-IR (homeostasis model assessment of insulin resistance), age, HbA1c, fasting blood glucose, selenium and GPX3 concentrations at baseline. We defined the onset of glucose intolerance by combining participants diagnosed with type 2 diabetes and those with impaired glucose tolerance.

**Figure 3:**
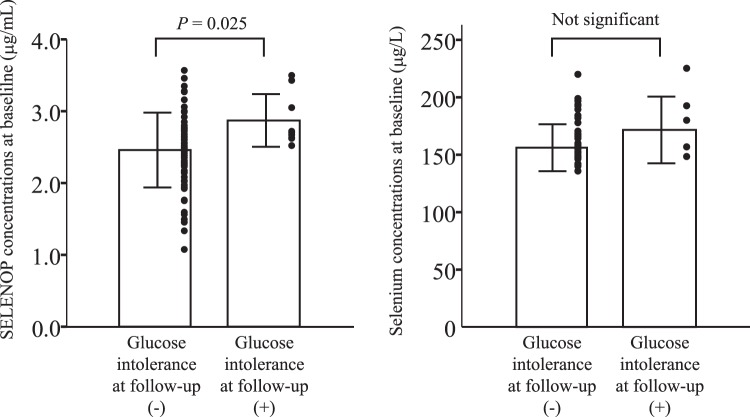
Serum concentrations of SELENOP and selenium at baseline in people with or without the development of glucose intolerance at follow up. We defined the onset of glucose intolerance by combining participants diagnosed with type 2 diabetes and those with impaired glucose tolerance. Data represent the means ± SD.

We also performed receiver operating characteristic (ROC) curve analysis to evaluate the sensitivity and specificity of different baseline variables on the prediction of the onset of glucose intolerance in 4-years follow-up (Fig. 2, Table 6). We defined the onset of glucose intolerance by combining the participants newly-diagnosed with impaired glucose tolerance or those with type 2 diabetes at 4-year follow up. As shown in Table 6, SELENOP concentrations at baseline had statistically significant prediction accuracy (Area under the curve = 0.723, *P* = 0.031), whereas baseline waist circumference, HOMA-IR, age, HbA1C, fasting plasma glucose, selenium, and GPX3 activity were not useful for predicting future risk of glucose intolerance. Additionally, concentrations of SELENOP at baseline, but not of selenium, were higher in participants who developed glucose intolerance over the 4-year follow-up compared with participants who did not develop glucose intolerance (Fig. 3).

**Table 6:** Area under the curve of various test variables in 2008 predicting to the onset of glucose intolerance at 4- year follow-up.

Test Result Variable(s)	Area under the curve	*P*
SELENOP in 2008	0.826	0.019*
Waist circumference in 2008	0.677	0.202
HOMA-IR in 2008	0.590	0.518
Age in 2008	0.500	1.000
HbA1c in 2008	0.656	0.259
Fasting blood glucose in 2008	0.482	0.897
Selenium in 2008	0.669	0.222
GPX3 in 2008	0.282	0.116

^*^p < 0.05.

See Table 1 for abbreviations.

## Discussion

The current study demonstrates that elevation of circulating hepatokine SELENOP is independently connected to future onset of hyperglycemia in healthy participants. We have previously reported that injection of physiological doses of SELENOP induces hyperglycemia in normal mice^18^. These results indicate that SELENOP functions as a hepatokine that induces hyperglycemia in rodents. To date, however, no clinical prospective studies were available regarding the relationship between elevation of circulating SELENOP and hyperglycemia. The current data suggest that an excess of circulating SELENOP plays a causal role in the development of aging-related impairment of glucose metabolism in humans.

SELENOP concentrations at baseline showed significant and negative correlation to insulinogenic index, but not to HOMA-IR in the current healthy participants. We have previously reported that hepatic gene expression for *SELENOP* correlated negatively with metabolic clearance rate, a representative marker of systemic insulin sensitivity measured by hyperinsulinemic-euglycemic glucose clamp experiments, in patients with type 2 diabetes^1^. This suggests that hepatic overproduction of SELENOP coexists with systemic insulin resistance in type 2 diabetic condition. At first glance, the current results may seem to be contradictory. However, more recently, we have revealed that treatment with physiological doses of SELENOP reduces production and secretion of insulin in normal mice^18^. This report raises the possibility that an excess of circulating SELENOP induces both impaired insulin signaling in the peripheral tissue and decreased insulin secretion in the pancreas. Because HOMA-IR is a marker of insulin resistance based on hyperinsulinemia^16^, the direct inhibitory action of SELENOP on pancreatic insulin secretion could produce a seeming discrepancy between SELENOP and HOMA-IR in the current non-diabetic population.

The current data show that circulating levels of SELENOP, but not of selenium, were connected to the future onset of hyperglycemia in a general Japanese population. Growing evidence suggests that selenium exposure may increase the risk of type 2 diabetes in humans^19–21^. A recent meta-analysis revealed a direct association between blood levels of selenium and risk of diabetes with a clear and linear trend across different countries and study designs^22^. First, the nonsignificant effects of selenium on glucose metabolism in the current study may be due to very small sample size and short observation period. Second, excessive amounts of SELENOP could further dysregulate glucose metabolism beyond the effects of increased levels of selenium, because SELENOP itself possesses the direct anti-oxidative enzyme activity independent of its selenium transport capacity^23,24^. Additional large scale clinical studies are needed to determine the contributions of SELENOP and selenium to the onset of glucose intolerance in general populations.

Our observation of elevated SELENOP during the follow-up period and the positive correlation between SELENOP and age at baseline raise the possibility that aging increases circulating levels of SELENOP in the general Japanese population. Similarly, a previous clinical study showed a positive correlation between SELENOP concentrations and age in Danish people^25^. The molecular mechanism by which aging increases SELENOP concentrations in the blood is currently unknown, but our study suggests that increased circulating SELENOP might contribute to aging-associated glucose intolerance in humans.

Our current study shows that SELENOP concentrations at baseline were connected with future post-load plasma glucose in the male participants, whereas they were connected with future fasting plasma glucose in the female participants. In general, postprandial hyperglycemia is strongly affected by skeletal muscle insulin resistance and defects in pancreatic insulin secretion, whereas fasting hyperglycemia is affected by hepatic insulin resistance^26,27^. The current results lead us to speculate that sexual dimorphism is present in the main target organs of SELENOP in humans. The complexity of sexual dimorphism in selenium metabolism and selenoprotein regulation has been reported in several review articles^28,29^. Further large scale clinical studies are needed to elucidate the sexual dimorphism in the actions of SELENOP on glucose metabolism.

Overproduction of SELENOP and the development of metabolic disorders might reinforce each other in a vicious cycle. Because insulin exerts the inhibitory effects on gene expression for *SELENOP* in the hepatocytes^1,30,31^, impaired insulin action in fatty liver or type 2 diabetes may up-regulate SELENOP production in the liver. Because endoplasmic reticulum (ER) stress increases SELENOP production in the hepatocytes^32^, high fat diet-induced ER stress in the liver may promote SELENOP production. Thus, certain metabolic disorders such as type 2 diabetes might increase circulating SELENOP. However, the hypothesis that overproduction of SELENOP contributes to the pathogenesis in metabolic disorders is supported by the current findings that increased blood levels of SELENOP predict future onset of hyperglycemia in humans.

We have previously shown that SELENOP impairs insulin signal transduction in the cultured hepatocytes in an autocrine/paracrine manner by up-regulation of protein phosphatase 2C (PP2C) and inactivation of adenosine monophosphate-activated protein kinase (AMPK)^1^. Additionally, SELENOP acts on the cultured myotubes through the receptor LRP1 to induce insulin resistance^5^. Taken together with these early reports, the current findings raise the possibility that pharmacological or lifestyle interventions against increased circulating levels of SELENOP could improve glucose metabolism by attenuating systemic insulin resistance in humans.

Selenoproteins other than SELENOP, such as glutathione peroxidase 1 (GPX1) and selenoprotein S, are known to play a major role in regulation of glucose metabolism^33,34^. For example, overexpression of GPX1 was reported to exhibit insulin resistance and obesity in mice^35^. Increased circulating levels of SELENOP might contribute to future hyperglycemia by inducing the other selenoproteins such as GPX1. Further animal or cellular studies are needed to determine the selenoproteins that function as downstream targets of SELENOP.

A strength of the current study is the selective measurement of full length form of SELENOP by using two types of monoclonal antibodies, one recognizing N terminal domain of SELENOP and another recognizing C-terminal side^36^. A previous report showed that plasma kallikrein proteolysis produces N- and C-terminal fragments of SELENOP from full length form of human SELENOP^37^. At present, several kinds of measurement kits for circulating human SELENOP are commercially available. However, many of the manufacturers did not indicate the selectivity of those commercial kits against full length form of SELENOP. We have previously reported that treatment with full length form of SELENOP impairs insulin signal transduction in cultured hepatocytes^1^, but the function of fragments forms of SELENOP in glucose metabolism has not yet been established. When assessing the significance of circulating SELENOP in glucose metabolism in humans, it might be desirable to consider the difference between the full length form and fragments forms of SELENOP.

We have not yet determined normal values of serum SELENOP in Japanese populations when applying our method specifically to the full-length form of SELENOP^36^. There are several difficulties in determining normal values of SELENOP. First, SELENOP concentrations increase gradually with aging. Second, males show higher concentrations of SELENOP than females. Third, SELENOP concentrations increase in relation to hyperglycemia. Thus, to calculate our tentative normative values, we selected participants with normal glucose tolerance, and an age less than 50 years, from among the study population. The average serum SELENOP concentration in males and females was 2.4 ± 0.7 and 2.2 ± 0.4, respectively. However, additional blood sampling on large numbers of healthy human subjects is necessary to determine normative values of circulating SELENOP, stratified by gender and age, in the Japanese general population.

We previously reported that serum concentration of SELENOP was approximately 5.1 μg/mL in normal subjects, when measured by sandwich enzyme-linked immunosorbent assay using two kinds of antibodies against N-terminal domains of SELENOP^1^. However, in the current study, average concentration of serum SELENOP was 2.5 μg/mL (Table 1). The present values appear to be lower compared with our previous report. This is explained by the fact that we currently used sol particle homogeneous immunoassay using two monoclonal antibodies against SELENOP, one recognizing the N-terminal domain and the other recognizing the C-terminal domain, to measure serum concentrations of full length form of SELENOP selectively^38^. Because SELENOP has kallikrein cutting sites in its central part, the N-terminal and C-terminal fragments of SELENOP are derived from full length SELENOP by the proteolysis of blood kallikrein^37^. The actions of short fragments of SELENOP on glucose metabolism are still unknown, but the current results suggest that increased levels of full length form of SELENOP are connected to future hyperglycemia in humans.

Another strength of the current study is that we evaluated glucose metabolism by performing 75-g OGTT in all of the study subjects at both baseline and 4-year follow-up. We thereby precisely evaluated relationship of circulating SELENOP with both insulin resistance and insulin secretion.

In the current study, 12% of participants developed either type 2 diabetes or impaired glucose tolerance during the 4-year follow-up period. It appears that the incidence rate of glucose intolerance is very high in the current population. However, several clinical papers on general Japanese populations reported that the incidence rate of prediabetes was 15–23%^39,40^. The high incidence rate of glucose intolerance in Japanese populations is thought be connected with a low capacity for insulin secretion^40,41^. Therefore, we believe that the incidence rate of glucose intolerance of the current population is consistent with the general Japanese population.

An early paper showed that SELENOP concentrations were unchanged in pregnant women with gestational diabetes compared with those without gestational diabetes^10^. In that paper, the average HbA1c in pregnant women with gestational diabetes was 5.4%. Unchanged levels of SELENOP could be explained by the fact that severity of glucose intolerance was mild in the women with gestational diabetes. Additionally, differences in the methods used to measure SELENOP concentrations could affect the result in people with gestational diabetes^14^.

A limitation of the current study includes small sample sizes of the human subjects. In particular, only one human subject developed type 2 diabetes at 4-year follow up, probably due to small sample sizes. For this reason, we performed ROC analysis on the prediction of glucose intolerance by combining people diagnosed with type 2 diabetes and those with impaired glucose tolerance. Additional clinical studies on a larger number of samples are required to assess whether elevation of circulating SELENOP is involved in the development of type 2 diabetes.

In the current study, HbA1c and fasting plasma glucose concentrations showed significant positive correlations at both time points, baseline and 4-year follow-up (data not shown). However, HbA1c values were unchanged before and after the follow-up period, although the average fasting plasma glucose concentrations increased significantly by 4.6 mg/dl. According to an early clinical paper^42^, an increase in fasting plasma glucose of 4.6 mg/dl corresponds to an increase in HbA1c of only 0.07%. Hence, a very small increase in fasting plasma glucose concentrations during the follow up period might have resulted result in insignificant changes in HbA1c in the current study.

Both fasting plasma glucose concentrations and HbA1c are well-known predictors of diabetes mellitus. Growing evidence shows that the predictive value of fasting plasma glucose for diabetes is nearly equal to that of HbA1c^43,44^. However, in the current study, ROC analysis of the prediction of future glucose intolerance showed that the area under the curve for HbA1c was somewhat larger than that of fasting plasma glucose (Fig. 1 and Table 6). Elevation of fasting plasma glucose levels reflects acute dysregulated glucose metabolism, whereas elevation of HbA1c reflects chronic hyperglycemia including postprandial glucose response^45^. This difference in the sensitivity of the two predictors to long-term hyperglycemia could produce the result in the current study.

In conclusion, the current data reveal that elevation of circulating SELENOP is positively and independently associated with the future onset of glucose intolerance in a general population. Further studies are needed to determine whether interventions on SELENOP prevent the deterioration of glucose tolerance in humans.

## Methods

### Ethics Statement

All patients provided written informed consent for the current study. The experimental protocol was approved by the Medical Ethics Committee of Kanazawa University (Approval No. 2011-049), and the study was conducted in accordance with the Declaration of Helsinki.

### Human study

Human subjects were 76 Japanese non-diabetic and non-pregnant participants who went to the Ishikawa Matto Central Hospital for a complete physical examination. The people with type 2 diabetes were excluded in this study. No patients received any oral hypoglycemic agents, such as pioglitazone or biguanide. All participants underwent a 75 g oral glucose tolerance test (75gOGTT) as part of the study, regardless of clinical indications such as pregnancy or increased body weight. First, 75gOGTT was performed in 2008. Serum concentrations of SELENOP in the fasting serum samples in 2008, which were stored frozen at −20 degree, were measured. 4 years later, the participants were performed 75-g OGTT again to assess the alteration of glucose tolerance in 2012. People with type 2 diabetes or impaired glucose tolerance were diagnosed on the basis of criteria established by an expert committee on the diagnosis and classification of diabetes mellitus^46^.

### Assays

Serum concentrations of full length SELENOP were measured by sol particle homogeneous immunoassay using two monoclonal antibodies, as we established previously^36,47^. The within- and between-day coefficients of variation ranged from 0.73% to 2.24% and 0.45% to 1.11%, respectively. Serum concentrations of selenium were measured by atomic absorption spectrophotometry^48^. GPX3 activities in serum were measured by kinetic assay as previously described^24,49^. In brief, test and references samples were prepared in 96 wells plate which contained 0.1 M Tris-Hcl (pH 8.0), 0.5 mM EDTA, 2 mM Reduced Glutathione, 1 U/ml of glutathione reductase, 0.2 mM NADPH and 10 μl of serum in a total volume of 200 μl. The mixture was preincubated at 37 °C for 3 minutes, measuring the background absorbance of 340 nm every 10 seconds. After that, the reaction was started by adding 2 μl of 7 mM tert-butyl hydrogen peroxide into each wells. The absorbance at 340 nm was then measured every 10 seconds for 5 minutes at 37 °C. The GPX3 activities were then calculated as the decrease in absorbance at 340 nm. After dividing by molar extinction coefficient of NADPH and the sample volume, GPX3 activities in the test serum were described as U/L.

The immunoenzymometric assays used for quantifying insulin were conducted with kits purchased from Tosoh (Shunan, Japan). The Insulinogenic Index was calculated as the ratio of the increment of plasma insulin (micro–international units per milliliter) to the increment in glucose (milligrams per deciliter) during the first 30 min of OGTT^15^. The HOMA-IR was calculated using the following formula: HOMA-IR = [fasting insulin (μU/ml) × fasting plasma glucose (mmol/L)]/22.5^16^.

Serum concentrations of selenium were measured by an atomic absorption spectrometer SpectrAA 240Z (Agilent Technologies, England)^50,51^.

### Statistical Analysis

Data are shown as means ± standard deviation. The relationships among individual variables were analyzed by Spearman’s simple correlation, and multiple regression using a forced entry manner procedure. Differences between the two groups were assessed using unpaired two-tailed Student t-tests in Fig. 3. Receiver operating characteristic (ROC) curve analysis was performed to graphically demonstrate the effectiveness of different result variables on the prediction of glucose intolerance at 4-years follow-up. The subjects with normal glucose tolerance test at 4-years follow-up were classified as negative actual state group and those with impaired glucose tolerance or diabetes were classified as positive actual state group. *P* value was calculated to test the Null hypothesis that regards 0.5 as area under the curve. The Japanese Windows edition of SPSS (ver. 11.0; SPSS, Inc., Chicago, IL, USA) was used for statistical analysis. *P* values < 0.05 were considered to indicate statistical significance.

## Electronic supplementary material

Supplementary Tables 1–5
