# Supplementary material for: Serum selenoprotein P, but not selenium, predicts future hyperglycemia in a general Japanese population
Source: Sci Rep. 2018 Nov 13;8:16727. doi: 10.1038/s41598-018-35067-2 (PMC6233151; doi:10.1038/s41598-018-35067-2)
Supplement: Supplementary file 1 — Supplementary Tables 1–5 [file 41598_2018_35067_MOESM1_ESM.doc]

**Serum selenoprotein P, but not selenium, predicts future hyperglycemia in a general Japanese population**

Swe Mar Oo^1^, Hirofumi Misu^1,2*^, Yoshiro Saito^3^, Mutsumi Tanaka^4^, Seiji Kato^4^, Yuki Kita^1^, Hiroaki Takayama^1^, Yumie Takeshita^1^, Takehiro Kanamori^1^, Toru Nagano^5^, Masatoshi Nakagen^5^, Takeshi Urabe^5^, Naoto Matsuyama^4^, Shuichi Kaneko^6^, and Toshinari Takamura^1*^

Supplementary Table 1. Linear regression analysis of baseline selenoprotein P concentrations with clinical parameters at the 4-year follow-up in the male participants (*n* = 42).

|  | SELENOP concentration at baseline | |
| --- | --- | --- |
| Parameters at 4-year follow-up | *p* value | *r* |
| Body weight | 0.553 | -0.094 |
| BMI (kg/m^2^) | 0.638 | -0.075 |
| Fasting plasma glucose (mg/dl) | 0.515 | 0.103 |
| Plasma glucose 30 min (mg/dl) | 0.150 | 0.226 |
| Plasma glucose 60 min (mg/dl) | 0.002** | 0.455 |
| Plasma glucose 120 min (mg/dl) | 0.071 | 0.281 |
| Fasting IRI (µU/ml) | 0.181 | -0.210 |
| IRI 30 min (µU/ml) | 0.106 | -0.253 |
| IRI 60 min (µU/ml) | 0.561 | 0.092 |
| IRI 120 min (µU/ml) | 0.589 | 0.086 |
| HbA1c (%) | 0.349 | 0.148 |
| HOMA-IR | 0.196 | -0.204 |
| Insulinogenic index | 0.119 | -0.257 |

***p* < 0.01.

BMI, body mass index; IRI, immunoreactive insulin; HbA1c, glycosylated hemoglobin; HOMA-IR, homeostasis model assessment of insulin resistance.

Supplementary Table 2. Linear regression analysis of baseline selenoprotein P concentrations with clinical parameters at the 4-year follow-up in the female participants (*n* = 34).

|  | SELENOP concentration  at baseline | |
| --- | --- | --- |
| Parameters at 4-year follow-up | *p* value | *r* |
| Body weight | 0.233 | -0.210 |
| BMI (kg/m^2^) | 0.715 | -0.065 |
| Fasting plasma glucose (mg/dl) | 0.001** | 0.546 |
| Plasma glucose 30 min (mg/dl) | 0.722 | 0.063 |
| Plasma glucose 60 min (mg/dl) | 0.256 | 0.200 |
| Plasma glucose 120 min (mg/dl) | 0.388 | 0.153 |
| Fasting IRI (µU/ml) | 0.839 | 0.036 |
| IRI 30 min (µU/ml) | 0.229 | -0.212 |
| IRI 60 min (µU/ml) | 0.608 | 0.091 |
| IRI 120 min (µU/ml) | 0.925 | -0.017 |
| HbA1c (%) | 0.230 | 0.211 |
| HOMA-IR | 0.513 | 0.116 |
| Insulinogenic index | 0.737 | -0.061 |

**p* < 0.05, ***p* < 0.01.

See Supplementary Table 1 for abbreviations.

Supplementary Table 3. Linear regression analysis of glutathione peroxidase 3 activity with clinical parameters at baseline.

|  | Serum glutathione peroxidase 3 activity  at baseline (*n* = 44) | |
| --- | --- | --- |
| Parameters at baseline | *p* value | *r* |
| Age | 0.125 | -0.235 |
| Body weight | 0.156 | -0.217 |
| BMI (kg/m^2^) | 0.062 | -0.283 |
| Waist Circumference (cm) | 0.036* | -0.316 |
| Fasting plasma glucose (mg/dl) | 0.266 | -0.172 |
| Plasma glucose 30 min (mg/dl) | 0.033* | -0.322 |
| Plasma glucose 60 min (mg/dl) | 0.483 | -0.108 |
| Plasma glucose 120 min (mg/dl) | 0.394 | -0.132 |
| Fasting IRI (µU/ml) | 0.823 | 0.035 |
| IRI 30 min (µU/ml) | 0.614 | -0.078 |
| IRI 60 min (µU/ml) | 0.933 | -0.013 |
| IRI 120 min (µU/ml) | 0.799 | 0.039 |
| HbA1c (%) | 0.706 | -0.058 |
| HOMA-IR | 0.911 | 0.017 |
| Insulinogenic index | 0.547 | 0.093 |
| AST (IU/L) | 0.743 | 0.051 |
| ALT (IU/L) | 0.190 | -0.201 |
| Triglyceride (mg/dl) | 0.164 | -0.213 |
| SELENOP (μg/mL) | 0.749 | 0.050 |
| Selenium (μg/L) (*n* = 44) | 0.636 | -0..073 |

**p* < 0.05, ***p* < 0.01.

BMI, body mass index; IRI, immunoreactive insulin; HbA1c, glycosylated hemoglobin; HOMA-IR, homeostasis model assessment of insulin resistance; AST, aspartate aminotransferase; ALT, alanine aminotransferease; SELENOP, selenoprotein P.

Supplementary Table 4. Linear regression analysis of glutathione peroxidase 3 activity at baseline with clinical parameters at the 4-year follow-up in all the participants (*n* = 76).

|  | Serum glutathione peroxidase 3 activity  at baseline (*n* = 44) | |
| --- | --- | --- |
| Parameters at 4-year follow-up | *p* value | *r* |
| Body weight | 0.005** | -0.414 |
| BMI (kg/m^2^) | 0.035* | -0.319 |
| Fasting plasma glucose (mg/dl) | 0.155 | -0.218 |
| Plasma glucose 30 min (mg/dl) | 0.143 | -0.224 |
| Plasma glucose 60 min (mg/dl) | 0.608 | -0.080 |
| Plasma glucose 120 min (mg/dl) | 0.415 | -0.126 |
| Fasting IRI (µU/ml) | 0.419 | 0.125 |
| IRI 30 min (µU/ml) | 0.883 | -0.023 |
| IRI 60 min (µU/ml) | 0.709 | -0.058 |
| IRI 120 min (µU/ml) | 0.776 | -0.044 |
| HbA1c (%) | 0.986 | -0.003 |
| HOMA-IR | 0.561 | 0.090 |
| Insulinogenic index | 0.086 | 0.271 |

**p* < 0.05, ***p* < 0.01.

BMI, body mass index; IRI, immunoreactive insulin; HbA1c, glycosylated hemoglobin; HOMA-IR, homeostasis model assessment of insulin resistance.

Supplementary Table 5. Linear regression analysis of increment of serum selenium during follow-up with clinical parameters at the 4-year follow-up.

|  | Increment of serum selenium concentrations  during follow-up  (*n* = 44) | |
| --- | --- | --- |
| Parameters at 4-year follow-up | *p* value | *r* |
| Body weight | 0.178 | -0.207 |
| BMI (kg/m^2^) | 0.461 | -0.114 |
| Fasting plasma glucose (mg/dl) | 0.595 | -0.082 |
| Plasma glucose 30 min (mg/dl) | 0.870 | 0.025 |
| Plasma glucose 60 min (mg/dl) | 0.634 | 0.074 |
| Plasma glucose 120 min (mg/dl) | 0.960 | -0.008 |
| Fasting IRI (µU/ml) | 0.361 | 0.141 |
| IRI 30 min (µU/ml) | 0.851 | 0.029 |
| IRI 60 min (µU/ml) | 0.508 | 0.103 |
| IRI 120 min (µU/ml) | 0.389 | 0.133 |
| HbA1c (%) | 0.972 | -0.006 |
| HOMA-IR | 0.502 | 0.104 |
| Insulinogenic index | 0.514 | 0.105 |

**p* < 0.05, ***p* < 0.01.

BMI, body mass index; IRI, immunoreactive insulin; HbA1c, glycosylated hemoglobin; HOMA-IR, homeostasis model assessment of insulin resistance.
